# Molecular Diagnostics in the Postgenomic Era

**DOI:** 10.3390/bioengineering11121259

**Published:** 2024-12-12

**Authors:** Petra Korać, Maja Matulić

**Affiliations:** Department of Biology, Division of Molecular Biology, Faculty of Science, University of Zagreb, Horvatovac 102, 10000 Zagreb, Croatia; petra.korac@biol.pmf.hr

Since the end of the 20th century, when the first eukaryotic organism was sequenced, genome sequencing as a technique has made incredible progress, and today new methods allow sequencing different genomes and genes at relatively low prices and in a short time. Besides enabling different techniques of cloning, overexpression, mutation, or gene exchange and recombination, as well as expression silencing, genome sequencing also enables the use of these techniques directly in diagnostics, either in the form of whole-genome sequencing, exome, RNAseq, or targeted gene sequencing. In parallel, there is a rapid development and search for molecular markers that can enable precise diagnostics and potential risk assessment. An especially rapidly developing field is the one of analyzing different markers in easily accessible human liquids, such as urine, blood serum, stool, etc., on one side, and detecting different RNA and DNA molecules, possibly released in the form of exosomes from disease-affected cells.

Besides RNA and DNA molecules, proteins and other biomolecules are also used in diagnostics. Today there is a wide range of various antibodies that detect specific marker molecules. Even more, mass spectrometry is also often used in molecular diagnostics.

Another field of research today is the investigation of the cell relationship with its environment, the influence of the microenvironment on diseased cells, and vice versa, especially in the context of tumor cells and their communication with infiltrating non-tumor cells. Therefore, there has been a great deal of effort to establish in vitro systems that could imitate the cell microenvironment in vivo. These systems usually involve 3D cell cultures, which could possibly give insight into normal conditions as well as conditions present in different diseases and possibly lead to new protocols for molecular diagnostics and therapies.

The field of molecular diagnostics has been broadening its range, introducing new diagnostic methods and targets, searching for new markers for different diseases, methods for detecting disease progression and therapy sensitivity, as well as ways to personalize the therapy. Therefore, the topics of the articles published in this Special Issue cover a wide range of molecular diagnostics, from the investigation of potential biomarkers to new methods and materials used in diagnostics. Also, as diagnostics do not necessarily involve only humans and human material, one article describes a method for detecting specific species of ants that is harmful to both people and invasive species in ecosystems.

Guseva and her collaborators [1] describe the design, characteristics, and potential use of BODIPY derivatives. BODIPY dyes are UV-absorbing small molecules that strongly emit in fluorescence wavelengths and therefore can be used for imaging different structures in cells and organisms. Their core structure can be modified in different ways to change their emitting wavelength and affinity toward different targets in the cell. The article published in this Special Issue describes a BODIPY biomarker that is covalently linked to the thioterpen, which has an affinity toward membranes and could possibly be used for nontoxic erythrocyte labeling.

The article written by Peterson et al. [2] presents miRNAs induced by radiation, which could be used as diagnostic biomarkers. They were detected on the model of human CGL cells, a nontumorigenic hybrid cell line obtained by fusion of fibroblasts and HeLa cells, after irradiation with 10–1000 mGy and different periods of postirradiation incubation. All miRNA samples were sequenced by next-generation sequencing, and potential differentially expressed miRNA, as well as their downstream targets, were analyzed by qRT-PCR. The authors selected miR-1228-3p and miR-758-5p as potential targets in radiation biodosimetry.

The article written by Krause et al. [3], “A systematic approach to diagnostic laboratory software requirements analysis”, presents the problem of growing needs for software adapted for validated procedures in the steadily growing number and complexity of molecular diagnostics techniques. They described the procedure for q-RT PCR in the form of a systematic requirement analysis for the implementation of the method in the laboratory. The first part of the process is understanding the state of the art in the field by conducting a literature review. The second part is a review of the standards and regulations as well as the technology, followed by a comparison of laboratory practice and the results of the literature review through a set of questions considering the samples, their number, origin, experiment settings, etc., and the analysis of laboratory practice, existing solutions, and requirements. The model includes, after the first step of observation, the creation of a conceptual model of the solution, the creation of a prototype of proof of concept implementation, and the evaluation of the conceptual model and implementation. In the results, the authors compared qPCR software features, including statistical methods, cluster analysis, and graphical presentations of data.

In the review article written by Matulić et al. [4], miRNA, as a tool in molecular diagnostics, is presented. The article covers the biogenesis of miRNA and the roles of specific miRNAs in different types of malignant tumors, from leukemia and lymphomas to brain tumors, lung, breast, and other types of carcinomas. Also, the role of miRNA in viral diseases linked with tumor development is presented. The review also presents methods for miRNA detection and their use in current molecular diagnostics and future perspectives. It was concluded that, although not yet in clinical use, different miRNAs are emerging markers with high potential in diagnostics and prognostics of disease development.

Finally, Kim and Koh [5] from Korea describe a fast and acute method for detection of the presence of imported fire ants that can be applied in the field. Fire ants (genus *Solenopsis*) include more than 200 species, and some of them can sting and cause urticaria with their venom. Some of them belong to invasive species, such as *Solenopsis invicta*, which spread from South America to eastern Asia and can be found in international ports during the quarantine period. Therefore, it is important to discern them from other red ants that do not belong to the Solenopsis genus but are morphologically similar. The method described is based on the loop-mediated isothermal amplification (LAMP). The authors describe new primers that hybridize with genomic DNA and require only 20–30 min for processing, instead of longer time periods required for other methods. LAMP uses 4–6 primers recognizing distinct regions of target DNA and DNA polymerase, which extend primers and amplify loop structures made by specific primers. The amplification reaction is exponential, and a huge amount of DNA products is produced in a short time, which can be visualized even by the naked eye using specific indicators, like DNA intercalating dyes. Also, the reaction requires only one temperature, usually between 55 and 70 °C, making it convenient for the fieldwork and much less requiring than PCR-based methods.

We hope that published articles will be useful and informative for the development of new diagnostic methods and applications based on given ideas.
